# Supplementary material for: A PPARγ transcriptional cascade directs adipose progenitor cell-niche interaction and niche expansion
Source: Nat Commun. 2017 Jun 26;8:15926. doi: 10.1038/ncomms15926 (PMC5490270; doi:10.1038/ncomms15926)
Supplement: Supplementary Information [file ncomms15926-s1.pdf]

Title of file for HTML: Peer Review File
Description:

Title of file for HTML: Supplementary Information
Description: Supplementary Figures and Table.

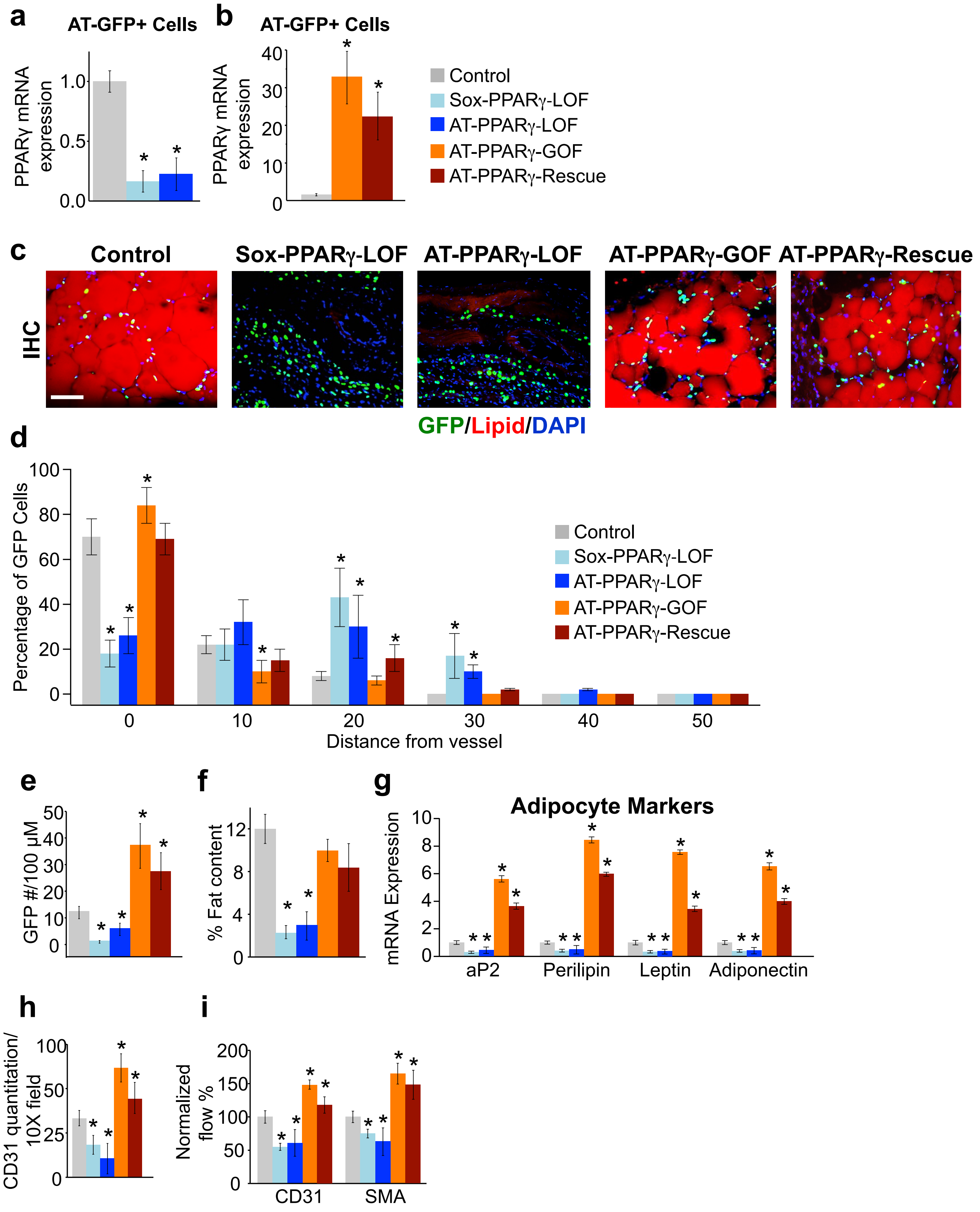

a
AT-GFP+ Cells
PPARγ mRNA expression
1.0
0.5
0.0
b
AT-GFP+ Cells
PPARγ mRNA expression
40
30
20
10
0
Control
Sox-PPARγ-LOF
AT-PPARγ-LOF
AT-PPARγ-GOF
AT-PPARγ-Rescue
c
Control
Sox-PPARγ-LOF
AT-PPARγ-LOF
AT-PPARγ-GOF
AT-PPARγ-Rescue
IHC
GFP/Lipid/DAPI
d
Percentage of GFP Cells
100
80
60
40
20
0
0
10
20
30
40
50
Distance from vessel
e
GFP #/100 μM
f
% Fat content
g
Adipocyte Markers
mRNA Expression
aP2
Perilipin
Leptin
Adiponectin
h
CD31 quantitation/ 10X field
i
Normalized flow %
CD31
SMA

Supplementary Fig. 1 PPARγ is not required for adipose depot developmental patterning

(**a,b**) AT-GFP+ cells were FACS isolated from AdipoTrak control (PPARγ^{tTA}; TRE-H2B-GFP), Sox-PPARγ-LOF (Sox2-Cre; PPARγ$^{f/tTA}$; TRE-H2B-GFP), AT-PPARγ-LOF (TRE-Cre; PPARγ$^{f/tTA}$; TRE-H2B-GFP), AT-PPARγ-GOF (PPARγ^{tTA}; TRE-PPARγ; TRE-H2B-GFP) and AT-PPARγ-Rescue (TRE-Cre; PPARγ$^{f/tTA}$; TRE-PPARγ; TRE-H2B-GFP) and mRNA expression of PPARγ was examined. Experiments were performed three times on 6 mice/ group. (**c**) Representative images of lipid (LipidTox) stained cyrosections from subcutaneous IGW depots from the GFP labeled mice described in (**a**). DAPI was used to visual nuclei and cell number. Scale Bar 100 μm. (**d**) Quantification of AT-GFP distance away from CD31/SMA+ blood vessels from sections described in Figure 1d. (**e**) Representative images of SVPs isolated from mice described in (**a**). AT-GFP number was quantified per 100-micron SVP. (**f**) Fat content from mice described in (**a**). (**g**) Quantitative RT-PCR analysis of adipocyte markers from mice described in (**a**). (**h**) Number of CD31+ cells were quantified from immunostained sections from subcutaneous IGW depots from denoted mice. (**i**) The SV compartment was isolated from mice described (**a**). Cells were stained with antibodies against CD31 and SMA and analyzed by flow cytometry. *$P<0.05$ unpaired t-test, two-tailed: mutants compared to control. Data are expressed as means ±s.em.

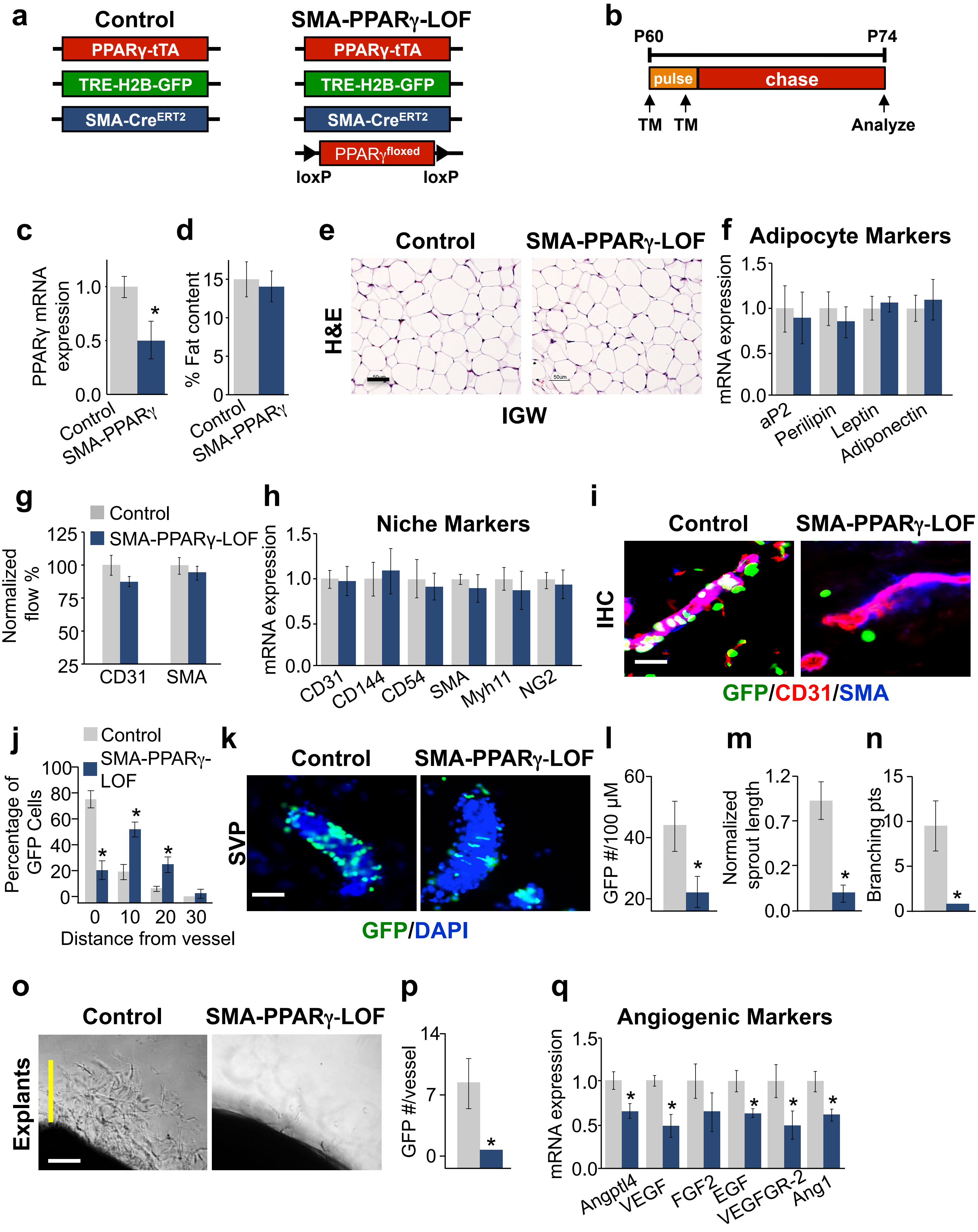
a
Control
PPARγ-tTA
TRE-H2B-GFP
SMA-CreERT2
SMA-PPARγ-LOF
PPARγfloxed
loxP
loxP
b
P60
P74
pulse
chase
TM
TM
Analyze
c
PPARγ mRNA expression
Control
SMA-PPARγ
d
% Fat content
Control
SMA-PPARγ
e
Control
SMA-PPARγ-LOF
H&E
IGW
f
Adipocyte Markers
mRNA expression
aP2
Perilipin
Leptin
Adiponectin
g
Control
SMA-PPARγ-LOF
Normalized flow %
CD31
SMA
h
Niche Markers
mRNA expression
CD31
CD144
CD54
SMA
Myh11
NG2
i
Control
SMA-PPARγ-LOF
IHC
GFP/CD31/SMA
j
Control
SMA-PPARγ-LOF
Percentage of GFP Cells
Distance from vessel
k
Control
SMA-PPARγ-LOF
SVP
GFP/DAPI
l
GFP #/100 μM
m
Normalized sprout length
n
Branching pts
o
Control
SMA-PPARγ-LOF
Explants
p
GFP #/vessel
q
Angiogenic Markers
mRNA expression
Angptl4
VEGF
FGF2
EGF
VEGFR-2
Ang1

Supplementary Fig. 2 Adult APC PPARγ expression is required for WAT niche expansion and APC-niche interaction

(**a**) Illustration of genetic alleles used to generate: AdipoTrak-SMA control (PPARγ$^{+/tTA}$; TRE-H2B-GFP; SMA-Cre^{ERT2}) and SMA-PPARγ-LOF (SMA-Cre^{ERT2}; PPARγ$^{f/tTA}$; TRE-H2B-GFP). (**b**) Diagram of experimental paradigm. Mice were administered one dose of tamoxifen (50 mg/ Kg) for two consecutive days. Mice were then chased for 14 days. Experiments were performed three times on 10 mice/group. (**c**) Quantitative RT-PCR analysis of PPARγ mRNA expression from SV cells after the 14 day chase. (**d**) Fat content of mice described in (**a**). (**e**) Representative images of H&E staining of subcutaneous IGW depots from mice described in (**a**). Scale Bar 50 μm. (**f**) Quantitative RT-PCR analysis of adipocyte markers from whole adipose tissue from mice described in (**a**). (**g**) The SV compartment was isolated from mice described (**a**). Cells were stained with antibodies against CD31 and SMA and were analyzed by flow cytometry for expression. (**h**) Quantitative RT-PCR analysis of niche markers (endothelial and mural cell markers) from the SV compartment of adipose depots from mice described in (**a**). (**i**) Representative images of CD31 (endothelial), SMA (mural) and AT-GFP staining of subcutaneous IGW depots from mice described in (**a**). (**j**) Quantification of AT-GFP distance away from CD31/ SMA+ blood vessels from sections described in (**i**). (**k**) Representative images of SVPs isolated from subcutaneous adipose depots from mice described in (**a**). AT-GFP locality was assessed and DAPI was used to visual nuclei. Scale Bar 100 μm. (**l**) Quantification of AT-GFP APC occupancy per 100 micron of SVPs. (**m-p**) Representative images of vascular sprouts from subcutaneous IGW depots from the mice described in (**a**). Sprout length (**m**), branching (**n**), sprouting (**o**), and AT-GFP sprout occupancy (**p**) were quantified. *P<0.01 unpaired t-test, two-tailed: mutant compared to control. Data are expressed as means ±s.e.m.

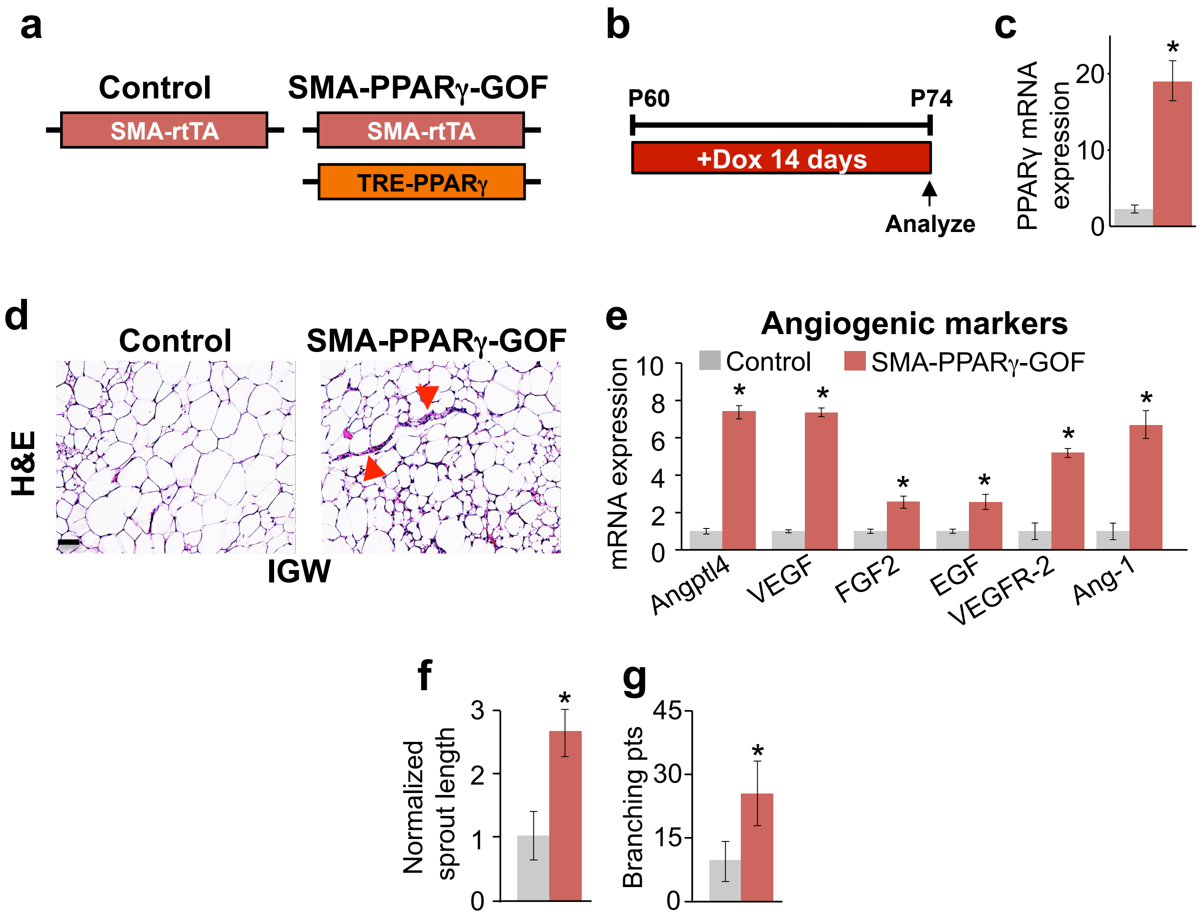

a
Control
SMA-PPARγ-GOF
SMA-rtTA
SMA-rtTA
TRE-PPARγ
b
P60
P74
+Dox 14 days
Analyze
c
PPARγ mRNA expression
20
10
0
*
d
Control
SMA-PPARγ-GOF
H&E
IGW
e
Angiogenic markers
Control
SMA-PPARγ-GOF
mRNA expression
10
8
6
4
2
0
Angptl4
VEGF
FGF2
EGF
VEGFR-2
Ang-1
f
Normalized sprout length
3
2
1
0
g
Branching pts
45
30
15
0

Supplementary Fig. 3 Adult APC PPARγ expression is required for WAT niche expansion and APC-niche interaction

(**a**) Illustration of genetic alleles used to generate: control (SMA-rtTA) and SMA-PPARγ-GOF (SMA-rtTA; TRE-PPARγ). (**b**) Diagram of experimental paradigm; mice were administered doxycycline (0.5 mg/ Kg) for 14 consecutive days. Experiments were performed three times on 8 mice/group. (**c**) Quantitative RT-PCR analysis of PPARγ mRNA expression from SV cells after the 14 day chase. (**d**) Representative images of H&E staining of subcutaneous IGW depots from mice described in (**a**). Red arrowheads indicate adipose tissue vasculature. Scale Bar 100 μm. (**e**) Quantitative RT-PCR analysis of angiogenic markers of SV cells from mice described in (**a**). (**f,g**) Vascular sprout length (**f**) and branching (**g**) were quantified from subcutaneous IGW depots from the mice described in (**a**) *$P<0.05$ unpaired t-test, two-tailed: mutant compared to control. Data are expressed as means ±s.e.m.

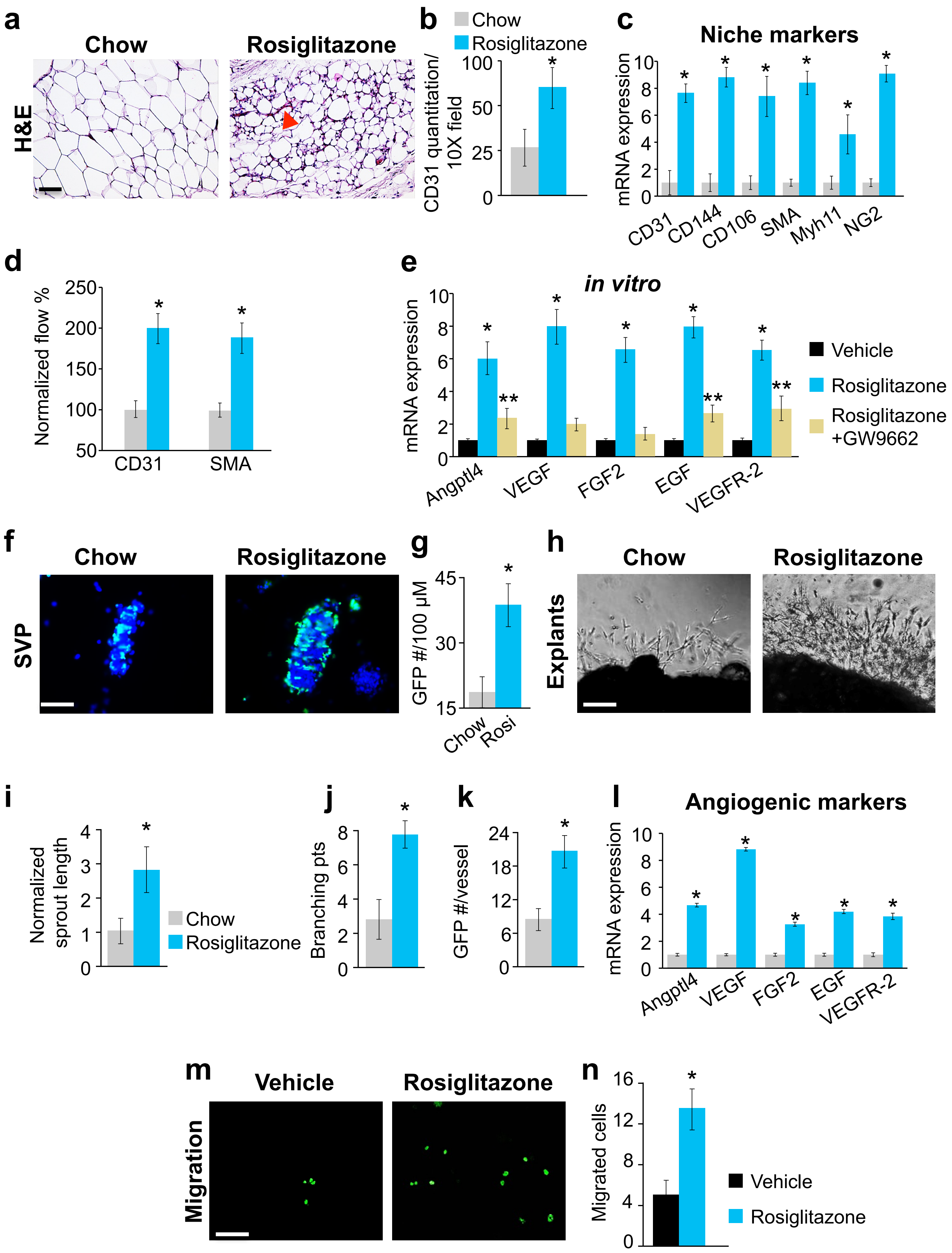

a
Chow
Rosiglitazone
H&E
b
Chow
Rosiglitazone
CD31 quantitation/ 10X field
c
Niche markers
mRNA expression
CD31
CD144
CD106
SMA
Myh11
NG2
d
Normalized flow %
CD31
SMA
e
in vitro
mRNA expression
Angptl4
VEGF
FGF2
EGF
VEGFR-2
Vehicle
Rosiglitazone
Rosiglitazone +GW9662
f
Chow
Rosiglitazone
SVP
g
GFP #/100 μM
Chow
Rosi
h
Chow
Rosiglitazone
Explants
i
Normalized sprout length
Chow
Rosiglitazone
j
Branching pts
k
GFP #/vessel
l
Angiogenic markers
mRNA expression
Angptl4
VEGF
FGF2
EGF
VEGFR-2
m
Vehicle
Rosiglitazone
Migration
n
Migrated cells
Vehicle
Rosiglitazone

Supplementary Fig. 4 TZD's stimulate WAT niche expansion and APC-niche interaction

(**a**) AdipoTrak control mice were administered normal chow or rosiglitazone supplemented chow (0.0075% diet) for 2 weeks and then analyzed for nichegenic potential and progenitor cell dynamics. Experiments were performed three times on 10 mice/group. Representative images of H&E staining of subcutaneous IGW depot. Red arrowhead indicates adipose tissue vasculature. Scale Bar 100 µm. (**b**) Number of CD31+ cells were quantified from immunostained sections from subcutaneous IGW depots from chow and rosiglitazone treated mice as described in (**a**). (**c**) Quantitative RT-PCR analysis of endothelial and mural cell markers from SV cells from mice described in (**a**). (**d**) Total SV cells were isolated from mice described in (**a**) and were stained with antibodies against CD31 and SMA. Positive cells were quantified by flow cytometry. (**e**) Total SV cells were pre-treated with vehicle or GW9662 (5 µM), a PPARγ antagonist for 15 mins, subsequently cells were treated with vehicle or rosiglitazone (1 µM) for 12 hours. Angiogenic gene expression was monitored. (**f,g**) Representative images of SVPs isolated from subcutaneous IGW depots from mice described in (**a**). SVPs were not supplemented with rosiglitazone *ex-vivo*. AT-GFP locality was assessed 12 hours after isolation (**f**). AT-GFP number were quantified per 100 micron SVP (**g**). Scale Bar 100 µm. (**h-k**) Representative images of vascular explants from subcutaneous IGW depots from mice described in (**a**). Vascular sprout length (**i**), branching points (**j**) and GFP progenitor occupancy (**k**) were quantified. Scale Bar 100 µm. (**l**) Quantitative RT-PCR analysis of angiogenic markers from subcutaneous IGW depots from mice described in (**a**). (**m,n**) SV cells were isolated from the mice described in (**a**) and AT-GFP+ were FACS isolated. Cells were cultured in modified Boyden transwell chambers. Cells were continually cultured with vehicle (DMSO) or rosiglitazone (1 µM). Migration was assessed 12 hours later (**m**) and AT-GFP number was quantified (**n**). Scale Bar 100 µm. *P<0.05 unpaired t-test, two-tailed: rosiglitazone treated compared to vehicle treated. **P<0.05 unpaired t-test, two-tailed: GW9662 + rosiglitazone compared to rosiglitazone alone. Data are expressed as means ± s.e.m.

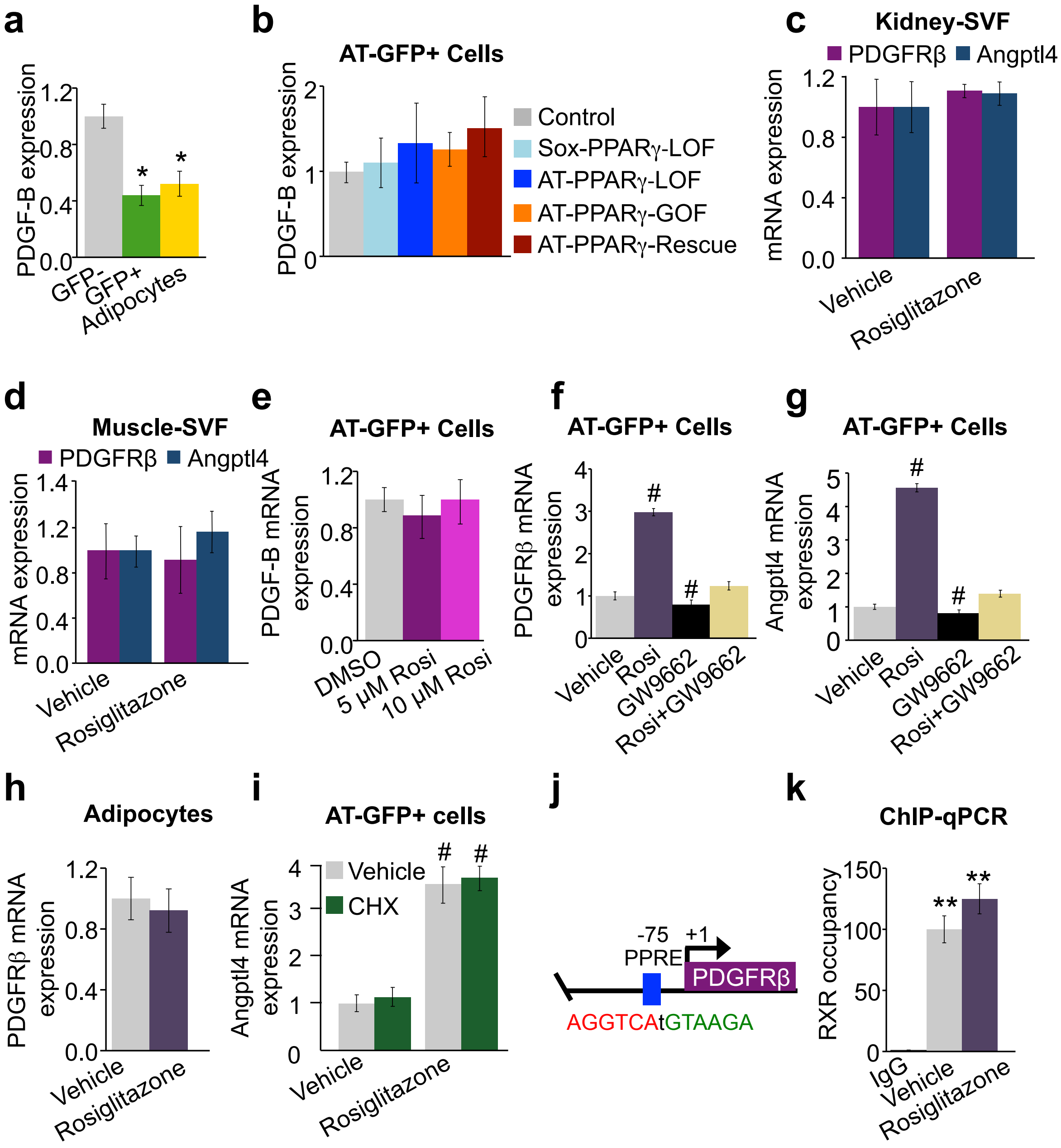
a
PDGF-B expression
1.2
0.8
0.4
0.0
*
*
GFP-
GFP+
Adipocytes
b
AT-GFP+ Cells
PDGF-B expression
2
1
0
Control
Sox-PPARγ-LOF
AT-PPARγ-LOF
AT-PPARγ-GOF
AT-PPARγ-Rescue
c
Kidney-SVF
PDGFRβ
Angptl4
mRNA expression
1.2
0.8
0.4
0.0
Vehicle
Rosiglitazone
d
Muscle-SVF
PDGFRβ
Angptl4
mRNA expression
1.4
1.2
0.8
0.4
0.0
Vehicle
Rosiglitazone
e
AT-GFP+ Cells
PDGF-B mRNA expression
1.2
0.8
0.4
0.0
DMSO
5 μM Rosi
10 μM Rosi
f
AT-GFP+ Cells
PDGFRβ mRNA expression
4
3
2
1
0
#
#
Vehicle
Rosi
GW9662
Rosi+GW9662
g
AT-GFP+ Cells
Angptl4 mRNA expression
5
4
3
2
1
0
#
#
Vehicle
Rosi
GW9662
Rosi+GW9662
h
Adipocytes
PDGFRβ mRNA expression
1.2
0.8
0.4
0.0
Vehicle
Rosiglitazone
i
AT-GFP+ cells
Angptl4 mRNA expression
4
3
2
1
0
Vehicle
CHX
#
#
Vehicle
Rosiglitazone
j
-75
+1
PPRE
PDGFRβ
AGGTCAtGTAAGA
k
ChIP-qPCR
RXR occupancy
150
100
50
0
**
**
IgG
Vehicle
Rosiglitazone

Supplementary Fig. 5 PPARγ transcriptionally controls niche formation, expansion and APC-niche interaction

(**a**) GFP- and AT-GFP+ cells were FACS isolated from AT-control mice (*n* = 6) and adipocytes were isolated by floatation. PDGF-B mRNA expression was measured. *P<0.01 compared to SV GFP negative cells. (**b**) AT-control, Sox-PPARγ-LOF, AT-PPARγ-LOF, AT-PPARγ-GOF and AT-PPARγ-Rescue were administered normal chow or rosiglitazone chow (0.0075%) for 7 days (*n* = 10). Subsequently, AT-GFP+ cells were FACS isolated and PDGF-B mRNA expression was measured. (**c,d**) Quantitative RT-PCR analysis of PDGFRβ and Angptl4 mRNA from SV cells isolated from kidney (**c**) and muscle (**d**) from mice described in (**b**). (**e**) GFP+ cells were FACS isolated from AT-GFP control mice (*n* = 6) and treated with denoted concentrations of rosiglitazone for 4 hours. mRNA expression of PDGF-B was measured. (**f,g**) Quantitative RT-PCR analysis of PDGFRβ and Angptl4 mRNA from FACS isolated AT-GFP+ from AT-control mice (*n* = 6) pre-treated with vehicle (DMSO) or GW9662 (5 µM) and then treated with vehicle (DMSO) or rosiglitazone (1 µM). #*P*<0.05 unpaired t-test, two-tailed: drug compared to vehicle. (**h**) Quantitative RT-PCR analysis of PDGFRβ mRNA expression from isolated adipocytes treated with vehicle (DMSO) or rosiglitazone (5 µM) for 4 hours. (**i**) Quantitative RT-PCR analysis of Angptl4 mRNA expression from FACS isolated AT-GFP+ cells from AT-control mice (*n* = 6) pretreated with cyclohexamide (10 µg/ml) and subsequently treated with vehicle (DMSO) or rosiglitazone. #*P*<0.05 unpaired t-test, two-tailed: drug treated compared to vehicle treated levels. (**j**) Schema of the PDGFRβ promoter and putative PPRE site. ChIP-qPCR analysis for RXR occupancy on the putative PPRE of the PDGFRβ promoter after treatment with vehicle (DMSO) or rosiglitazone (1 µM). ***P*<0.001 unpaired t-test, two-tailed: RXR IP compared to IgG. Data are expressed as means ±s.e.m.

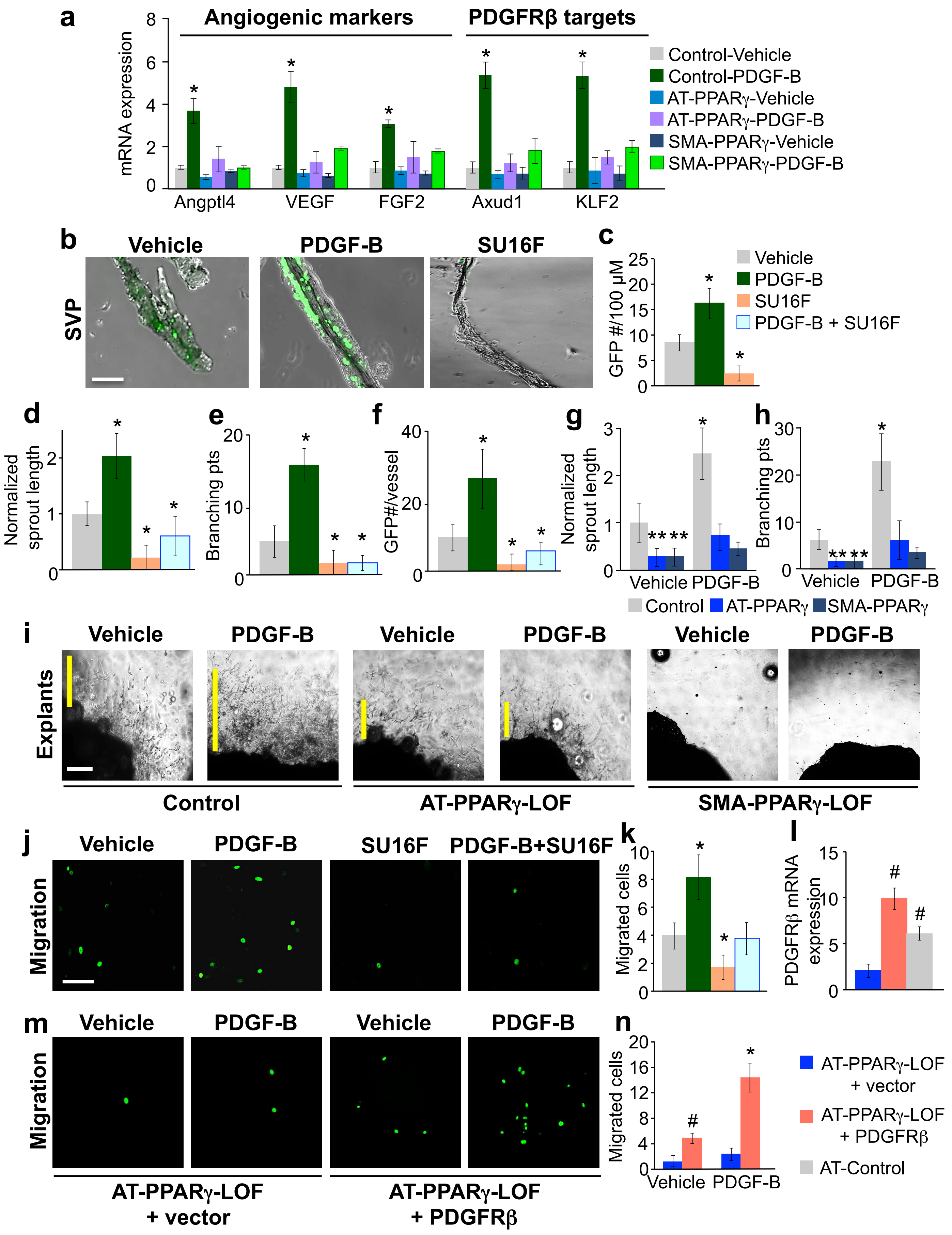
a
Angiogenic markers
PDGFRβ targets
mRNA expression
Angptl4
VEGF
FGF2
Axud1
KLF2
Control-Vehicle
Control-PDGF-B
AT-PPARγ-Vehicle
AT-PPARγ-PDGF-B
SMA-PPARγ-Vehicle
SMA-PPARγ-PDGF-B
b
Vehicle
PDGF-B
SU16F
SVP
c
GFP #/100 μM
Vehicle
PDGF-B
SU16F
PDGF-B + SU16F
d
Normalized sprout length
e
Branching pts
f
GFP#/vessel
g
Normalized sprout length
Vehicle
PDGF-B
h
Branching pts
Vehicle
PDGF-B
Control
AT-PPARγ
SMA-PPARγ
i
Vehicle
PDGF-B
Vehicle
PDGF-B
Vehicle
PDGF-B
Explants
Control
AT-PPARγ-LOF
SMA-PPARγ-LOF
j
Vehicle
PDGF-B
SU16F
PDGF-B+SU16F
Migration
k
Migrated cells
l
PDGFRβ mRNA expression
m
Vehicle
PDGF-B
Vehicle
PDGF-B
Migration
AT-PPARγ-LOF + vector
AT-PPARγ-LOF + PDGFRβ
n
Migrated cells
Vehicle
PDGF-B
AT-PPARγ-LOF + vector
AT-PPARγ-LOF + PDGFRβ
AT-Control

Supplementary Fig. 6 PDGFRβ stimulates WAT niche expansion and APC-niche interaction

(**a**) Quantitative RT-PCR analysis of angiogenic markers and PDGFRβ target genes from SV cells isolated from AT-Control, AT-PPARγ-LOF and SMA-PPARγ-LOF (*n* = 6) treated with vehicle (0.1% BSA) or PDGF-B (10 ng/ml) for 48 hours. *$P<0.01$ treated compared to vehicle. (**b,c**) Representative images of SVPs isolated from AT-control mice (*n* = 6) treated with vehicle, PDGF-B (10 ng/ml) or SU16F (5 μM) for 12 hours. APC-GFP fluorescence was imaged (**b**) and APC-GFP+ number was quantified per 100-micron SVP (**c**). Scale Bar 100 μm. *$P<0.01$ unpaired t-test, two-tailed: treated compared to vehicle. (**d-f**) Representative images of vascular sprouts from subcutaneous IGW explants from AT-control mice (*n* = 8). Explants were treated with vehicle, PDGF-B (10 ng/ml), SU16F (5 μM), or PDGF-B (10 ng/ml) and SU16F (5 μM). Vascular sprout length (**d**), branching (**e**) and AT-GFP sprout occupancy (**f**) were quantified. *$P<0.01$ unpaired t-test, two-tailed: treated compared to vehicle. (**g-i**) Representative images of vascular sprouts from subcutaneous IGW explants of AT-control, AT-PPARγ-LOF and SMA-PPARγ-LOF mice (*n* = 6) treated with vehicle or PDGF-B (10 ng/ml). Vascular sprout length (**g**), branching (**h**), and sprouting (**i**) were quantified. *$P<0.01$ unpaired t-test, two-tailed: treated compared to vehicle. **$P<0.01$ unpaired t-test, two-tailed: mutant compared to control. (**j,k**) Representative images of Boyden transwell migration chambers of FACS isolated APC-GFP+ cells from AT-GFP control mice (*n* = 6) pre-treated with vehicle or PDGF-B (10 ng/ml) for 48 hours prior to experimentation, migration was assessed 12 hours later (**j**). APC-GFP number was quantified (**k**). Scale Bar 100 μm. *$P<0.01$ unpaired t-test, two-tailed: treated compared to vehicle. (**l-n**) Representative image of modified Boyden transwell migration chambers of FACS isolated AT-GFP+ cells from AT-GFP control and AT-PPARγ-LOF mice (*n* = 6) transfected with PDGFRβ cDNA (**l**) and treated with vehicle or PDGF-B (10 ng/ml) for 12 hours (**m**). APC-GFP+ number was quantified (**n**). #$P<0.01$ unpaired t-test, two-tailed: compared to AT-PPARγ-LOF vector alone cells. *$P<0.01$ unpaired t-test, two-tailed: treated compared to vehicle. Scale Bar 100 μm. Data are expressed as means ±s.e.m.

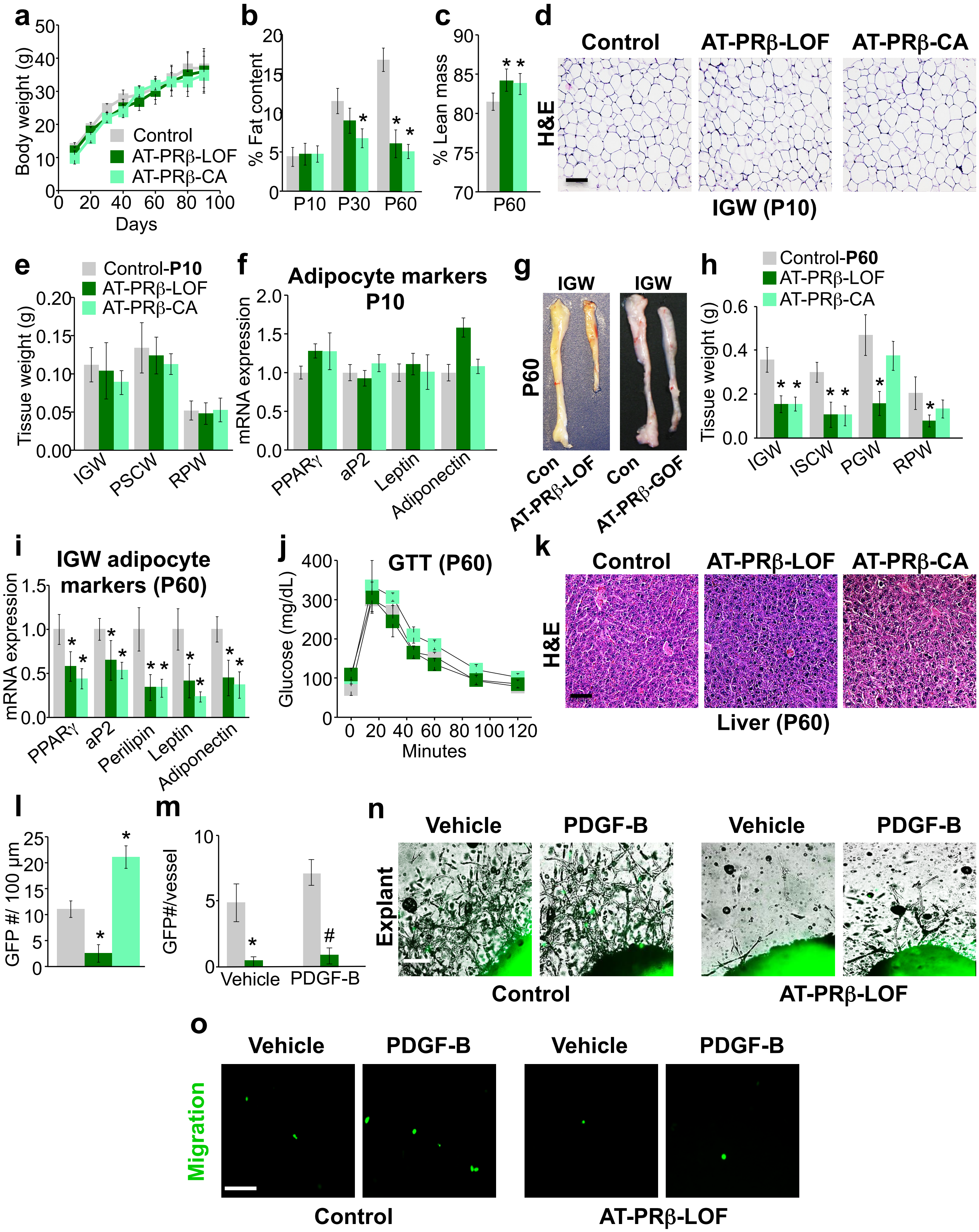
a
Body weight (g)
Days
Control
AT-PRβ-LOF
AT-PRβ-CA
b
% Fat content
P10 P30 P60
c
% Lean mass
P60
d
Control
AT-PRβ-LOF
AT-PRβ-CA
H&E
IGW (P10)
e
Control-P10
AT-PRβ-LOF
AT-PRβ-CA
Tissue weight (g)
IGW PSCW RPW
f
Adipocyte markers P10
mRNA expression
PPARγ aP2 Leptin Adiponectin
g
IGW IGW
P60
Con AT-PRβ-LOF Con AT-PRβ-GOF
h
Control-P60
AT-PRβ-LOF
AT-PRβ-CA
Tissue weight (g)
IGW ISCW PGW RPW
i
IGW adipocyte markers (P60)
mRNA expression
PPARγ aP2 Perilipin Leptin Adiponectin
j
GTT (P60)
Glucose (mg/dL)
Minutes
k
Control
AT-PRβ-LOF
AT-PRβ-CA
H&E
Liver (P60)
l
GFP #/ 100 µm
m
GFP#/vessel
Vehicle PDGF-B
n
Vehicle PDGF-B Vehicle PDGF-B
Explant
Control
AT-PRβ-LOF
o
Vehicle PDGF-B Vehicle PDGF-B
Migration
Control
AT-PRβ-LOF

Supplementary Fig. 7 PDGFRβ stimulates WAT niche expansion and APC-niche interaction

(**a-c**) Body weight (**a**), fat content (**b**), and lean mass (P60) (**c**) of AT-control, AdipoTrak PDGFRβ loss (AT-PDGFRβ-LOF) and constitutively active PDGFRβ (AT-PDGFRβ-CA) mice at denoted ages. Experiments were performed at least three times on 10 mice/group). (**d**) Representative images of H&E staining of subcutaneous IGW depots from mice described in (**a**) at P10. (**e**) Adipose tissue weights of mice described in (**a**) at P10. (**f**) Quantitative RT-PCR analysis of adipocyte markers from mice described in (**a**) at P10. (**g**) Representative photographs of subcutaneous IGW depots from mice described in (**a**) at P60. (**h,j**) Adipose tissue weight (**h**) and quantitative RT-PCR analysis of adipocyte markers (**i**) from mice described in (**a**) at P60. (**j**) Glucose tolerance test from mice described in (**a**) at P60. (**k**) Representative images of H&E staining of livers from mice described in (**a**) at P60. (**l**) Quantification of GFP number per 100-micron SVP. (**m,n**) Representative images of vascular sprouts from subcutaneous IGW explants from AT-control and AT-PDGFRβ-LOF mice treated with vehicle or PDGF-B. AT-GFP+ occupancy per vessel capillary (**m**) and vessel sprouting (**n**) were quantified. (**o**) Representative images of modified Boyden transwell migration chambers of FACS isolated AT-control and AT-PDGFRβ-LOF GFP+ cells pre-treated with PDGF-B for 48 hours. Migration was imaged 12 hours post plating. **P*<0.05 unpaired t-test, two-tailed: mutant compared to control. Scale Bar 100 μm. Data are expressed as means ±s.e.m.

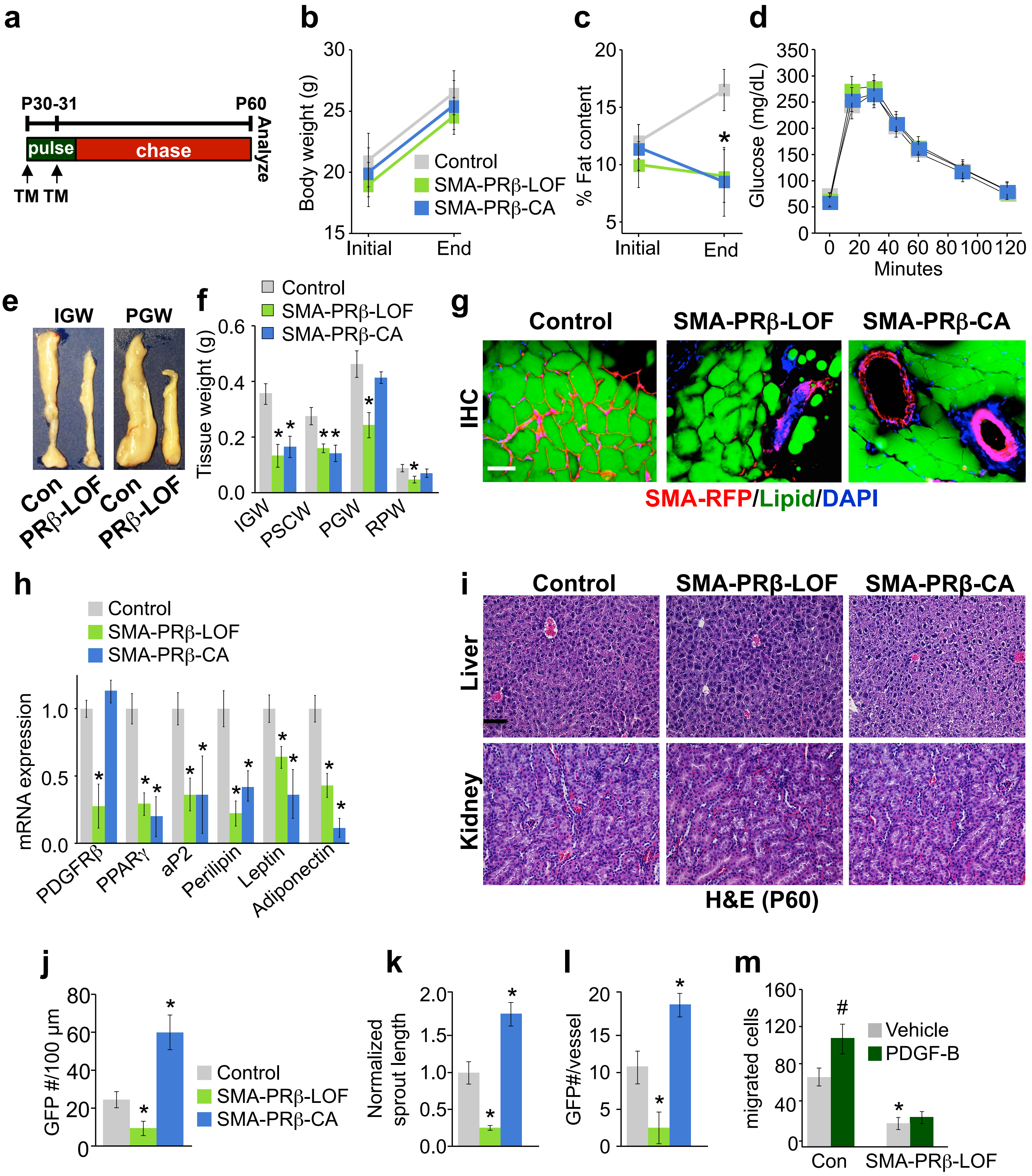

a
P30-31
P60
pulse
chase
Analyze
TM TM
b
Body weight (g)
Initial
End
Control
SMA-PRβ-LOF
SMA-PRβ-CA
c
% Fat content
Initial
End
d
Glucose (mg/dL)
Minutes
e
IGW
PGW
Con
PRβ-LOF
Con
PRβ-LOF
f
Tissue weight (g)
Control
SMA-PRβ-LOF
SMA-PRβ-CA
IGW
PSCW
PGW
RPW
g
Control
SMA-PRβ-LOF
SMA-PRβ-CA
IHC
SMA-RFP/Lipid/DAPI
h
Control
SMA-PRβ-LOF
SMA-PRβ-CA
mRNA expression
PDGFRβ
PPARγ
aP2
Perilipin
Leptin
Adiponectin
i
Control
SMA-PRβ-LOF
SMA-PRβ-CA
Liver
Kidney
H&E (P60)
j
GFP #/100 μm
Control
SMA-PRβ-LOF
SMA-PRβ-CA
k
Normalized sprout length
l
GFP#/vessel
m
migrated cells
Vehicle
PDGF-B
Con
SMA-PRβ-LOF

Supplementary Fig. 8 PDGFRβ stimulates APC-niche retention

(**a**) Diagram of experimental paradigm. AT-GFP-SMA-control, AT-GFP-SMA-PDGFRβ-LOF, and AT-GFP-SMA-PDGFRβ-CA mice were administered one dose of tamoxifen for two consecutive days, mice were analyzed 30 days later. Experiments were performed at least three times on 10 mice/group. (**b-f**) Body weight (**b**), fat content (**c**), GTT (**d**), depot photograph (**e**), adipose tissue weight (**f**), SMA adipocyte fate mapping images, and quantitative-RT-PCR analysis of adipocyte markers were assessed from mice described in (**a**). (**i**) Representative images of H&E staining of liver and kidney from mice described in (**a**). (**j**) Quantification of AT-GFP number per 100-micron SVP. (**k,l**) Quantification of vascular sprout length (**k**) and APC-GFP occupancy (**l**) of subcutaneous IGW explants from mice described in (**a**). (**m**) Quantification of modified Boyden transwell migration chambers assays of FACS isolated AT-control and AT-PDGFRβ-LOF GFP+ cells AT-SMA-control and AT-SMA-PDGFRβ-LOF GFP+ cells pre-treated with PDGF-B for 48 hours prior to migration assessment. Migration was assessed 12 hours post plating. *$P<0.05$ unpaired t-test, two-tailed: mutant compared to control. #$P<0.05$ unpaired t-test, two-tailed: treated compared to vehicle. Scale Bar 100 μm. Data are expressed as means ±s.e.m.

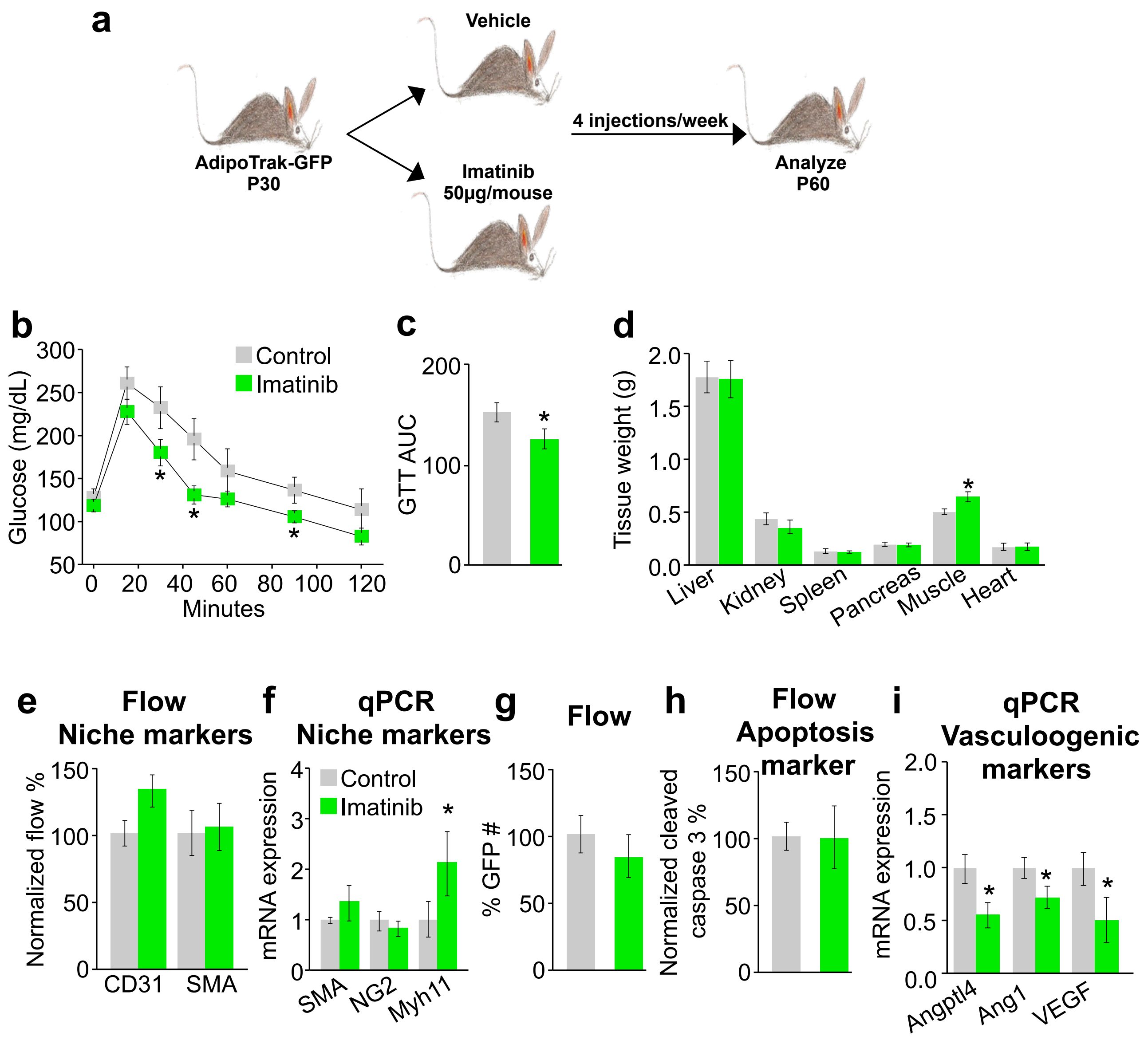

a
Vehicle
4 injections/week
AdipoTrak-GFP
P30
Imatinib
50µg/mouse
Analyze
P60
b
Control
Imatinib
Glucose (mg/dL)
Minutes
c
GTT AUC
d
Tissue weight (g)
Liver
Kidney
Spleen
Pancreas
Muscle
Heart
e
Flow
Niche markers
Normalized flow %
CD31
SMA
f
qPCR
Niche markers
mRNA expression
Control
Imatinib
SMA
NG2
Myh11
g
Flow
% GFP #
h
Flow
Apoptosis
marker
Normalized cleaved
caspase 3 %
i
qPCR
Vasculoogenic
markers
mRNA expression
Angptl4
Ang1
VEGF

Supplementary Fig. 9 Pharmacologically blocking PDGFRβ disrupts niche but improves glucose sensitivity

(**a**) Schema of experimental paradigm. AT-control male mice (P30) were administered vehicle (5%DMSO) or imatinib (50 ug/mouse) for four weeks. Experiments were preformed three times on 8 mice/group. (**b,c**) Glucose tolerance test (**b**) and calculated area under the curve (**c**) of mice described in (**a**) at the end of treatment. (**d**) Tissue weights of denoted organs from mice described in (**a**). (**e**) The SV compartment was isolated from subcutaneous adipose depots from mice described (**a**). Cells were stained with antibodies against CD31 and SMA and were analyzed by flow cytometry for expression. (**f**) Quantitative RT-PCR analysis of niche markers from SV cells isolated from adipose depots from mice described in (**a**). (**g**) Quantification of APC-GFP number by FACS from the total SV compartment of subcutaneous adipose depots from mice described (**a**). (**h**) Quantification of cleaved caspase 3 by flow cytometry from the total SV compartment from isolated subcutaneous adipose depots from mice described (**a**). (**i**) Quantitative RT-PCR analysis of angiogenic markers from the total SV compartment from subcutaneous adipose depots from mice described (**a**). *$P<0.01$ unpaired t-test, two-tailed: treated compared to vehicle. Data are expressed as means ±s.e.m.

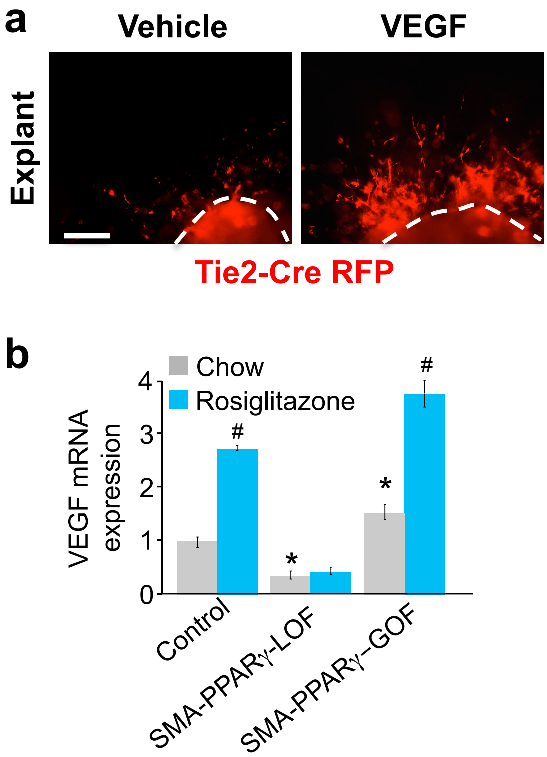
a
Vehicle
VEGF
Explant
Tie2-Cre RFP
b
Chow
Rosiglitazone
VEGF mRNA expression
0
1
2
3
4
#
*
#
*
Control
SMA-PPARγ-LOF
SMA-PPARγ-GOF

Supplementary Fig. 10 VEGF is a transcriptional target of PPARγ in APCs

(**a**) Representative fluorescent images of vascular explants from subcutaneous IGW depots from *Tie2-Cre; RosaR26R*^{RFP} male mice treated with vehicle or VEGF (n = 3). (**b**) Quantitative RT-PCR analysis of PDGFRβ mRNA expression from FACS isolated GFP+ cells from control, SMA-PPARγ-LOF, and SMA-PPARγ-GOF administered normal chow or rosiglitazone (0.0075% diet) for 7 days. *P<0.05 unpaired t-test, two-tailed: mutant compared control. #P<0.01 unpaired t-test, two-tailed: treated compared to vehicle. Scale Bar 100 μm. Data are expressed as means ±s.e.m.

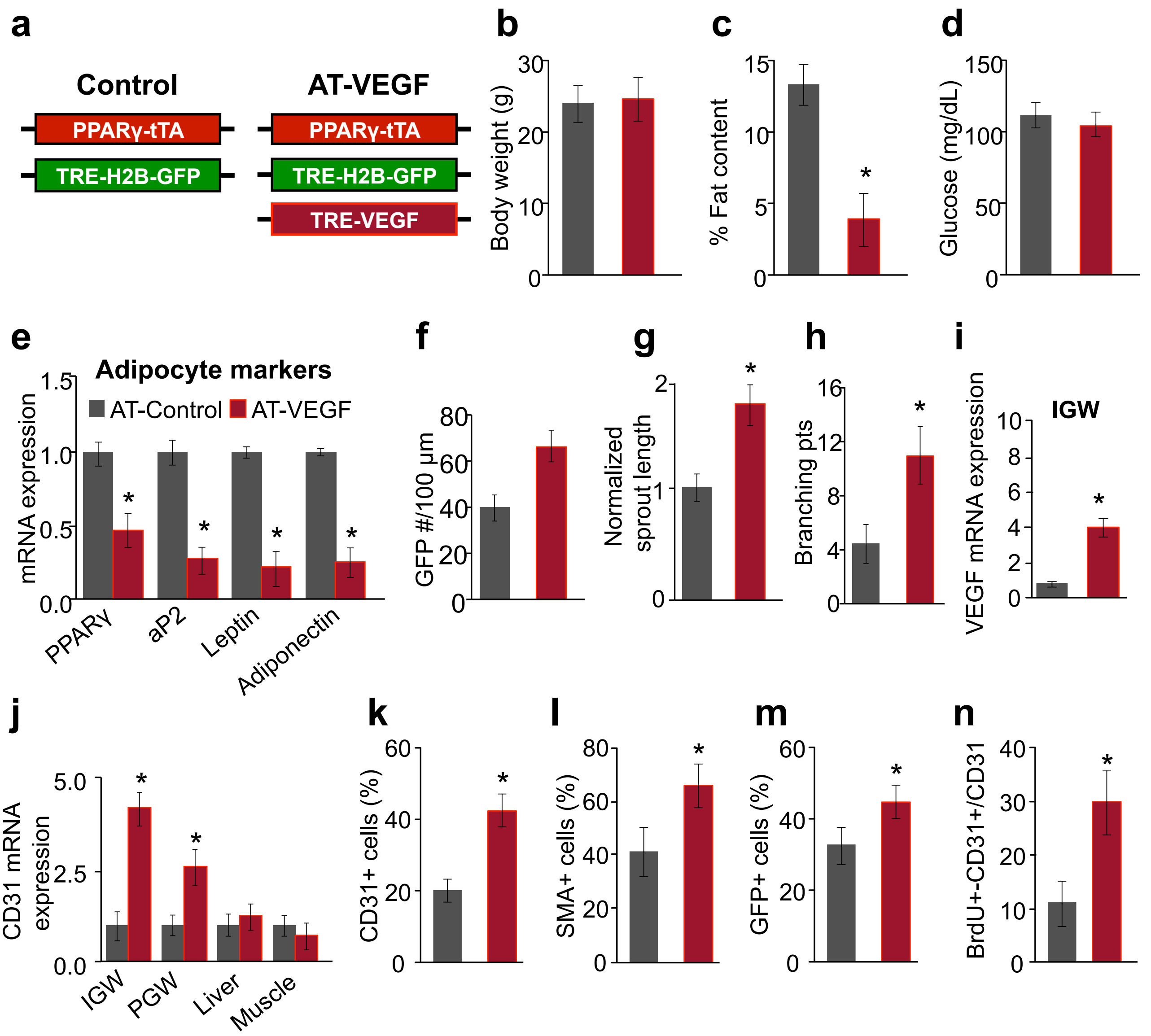

a
Control
PPARγ-tTA
TRE-H2B-GFP
AT-VEGF
PPARγ-tTA
TRE-H2B-GFP
TRE-VEGF
b
Body weight (g)
c
% Fat content
d
Glucose (mg/dL)
e
Adipocyte markers
AT-Control
AT-VEGF
mRNA expression
PPARγ
aP2
Leptin
Adiponectin
f
GFP #/100 μm
g
Normalized sprout length
h
Branching pts
i
IGW
VEGF mRNA expression
j
CD31 mRNA expression
IGW
PGW
Liver
Muscle
k
CD31+ cells (%)
l
SMA+ cells (%)
m
GFP+ cells (%)
n
BrdU+-CD31+/CD31

Supplementary Fig. 11 VEGF stimulates nichegenesis but blocks fat formation

(**a**) Diagram of genetic strategy: AT-control and AT-VEGF (*PPARγ$^{+/tTA}$; TRE-VEGF; TRE-H2B-GFP*). Experiments were performed three times on 10 mice/group. (**b-e**) Mice descried in (a) were analyzed at three-months of age for body weight (**b**), fat content (**c**), random sera glucose (**d**), and quantitative RT-PCR analysis of adipocyte markers (**e**). (**f**) Quantification of APC-GFP number per 100-micron SVP. (**g,h**) Quantification of vascular spout length (**g**) and branching (**h**) from subcutaneous IGW depots from mice described in (**a**). (**i,j**) Quantitative RT-PCR analysis of VEGF (**i**), and CD31 (**j**) from denoted tissues from mice described in (**a**). (**k-n**) Total SV cells were isolated from subcutaneous IGW depots from mice described in (**a**). Cells were stained and examined for CD31 (**k**), SMA (**l**) and APC-GFP (**m**) positive cells or SV cells were examined for BrdU and CD31+ cells by flow cytometry (**n**). *P<0.01 unpaired t-test, two-tailed: mutant compared to control. Data are expressed as means ±s.e.m.

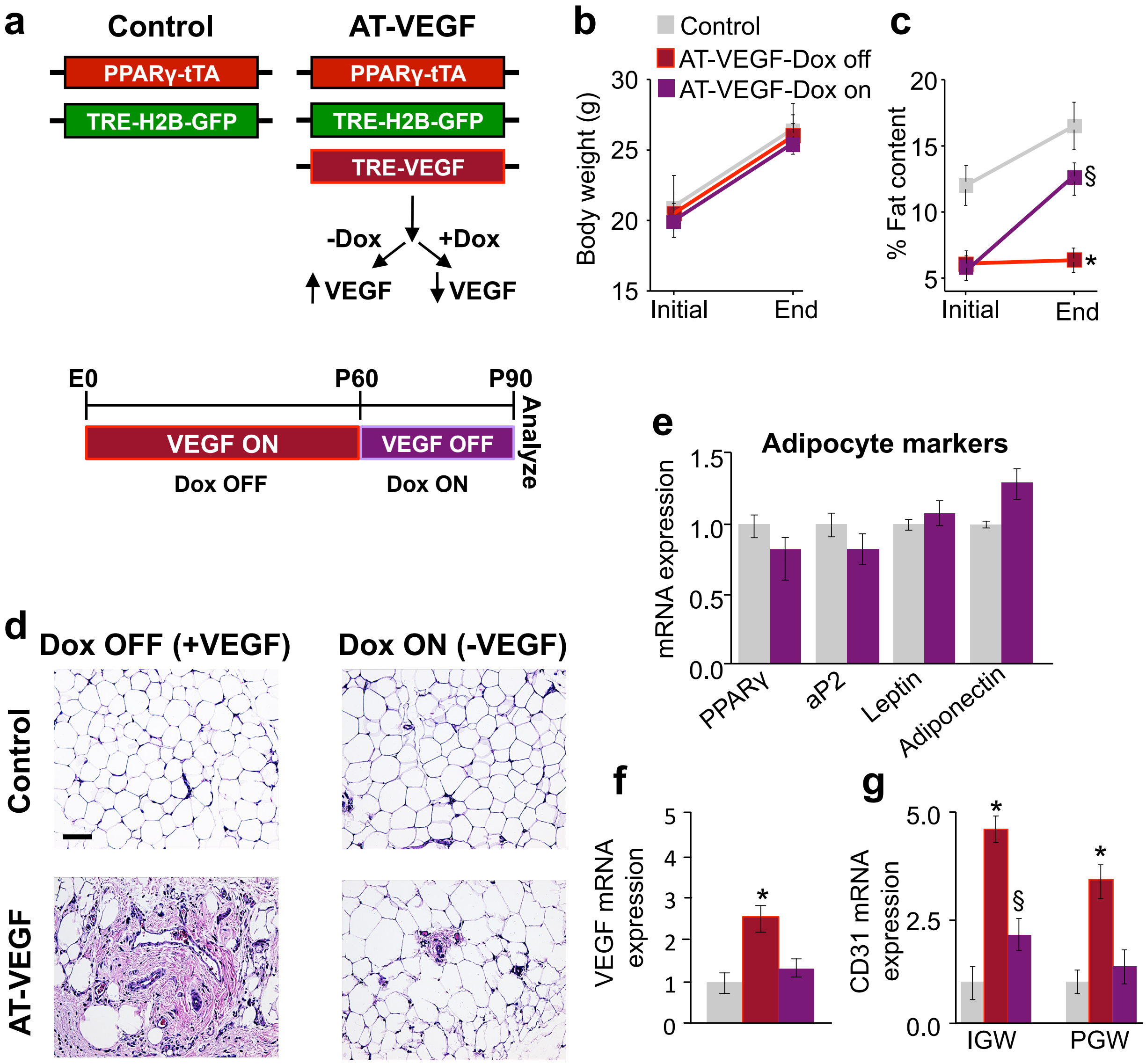

a
Control
AT-VEGF
PPARγ-tTA
TRE-H2B-GFP
PPARγ-tTA
TRE-H2B-GFP
TRE-VEGF
-Dox
+Dox
VEGF
VEGF
E0
P60
P90
Analyze
VEGF ON
VEGF OFF
Dox OFF
Dox ON
b
Control
AT-VEGF-Dox off
AT-VEGF-Dox on
Body weight (g)
30
25
20
15
Initial
End
c
% Fat content
20
15
10
5
§
*
Initial
End
d
Dox OFF (+VEGF)
Dox ON (-VEGF)
Control
AT-VEGF
e
Adipocyte markers
mRNA expression
1.5
1.0
0.5
0.0
PPARγ
aP2
Leptin
Adiponectin
f
VEGF mRNA expression
5
4
3
2
1
0
*
g
CD31 mRNA expression
5.0
2.5
0.0
*
§
*
IGW
PGW

Supplementary Fig. 12 Suppressing VEGF expression in APCs restores niche function and promotes fat formation.

(**a**) Diagram of genetic strategy and experimental paradigm: AT-control and AT-VEGF were maintained off Dox until two months of age and then administered Dox for 30 days. Experiments were performed twice on 8 mice/group. (**b,c**) Body weight (**b**) and fat content (**c**) of mice described in (**a**) were assessed. (**d**) Representative images of H&E staining of IGW depots from mice described in (**a**). (**e-g**) Quantitative RT-PCR analysis of adipocyte markers (**e**), VEGF (**f**), and CD31 (**g**) mice described in (**a**). *P<0.001 unpaired t-test, two-tailed: No Dox mutant compared to control mice. §P<0.05 unpaired t-test, two-tailed: Dox suppressed mutant compared to control mice. Scale Bar 100 μm. Data are expressed as means ±s.e.m.

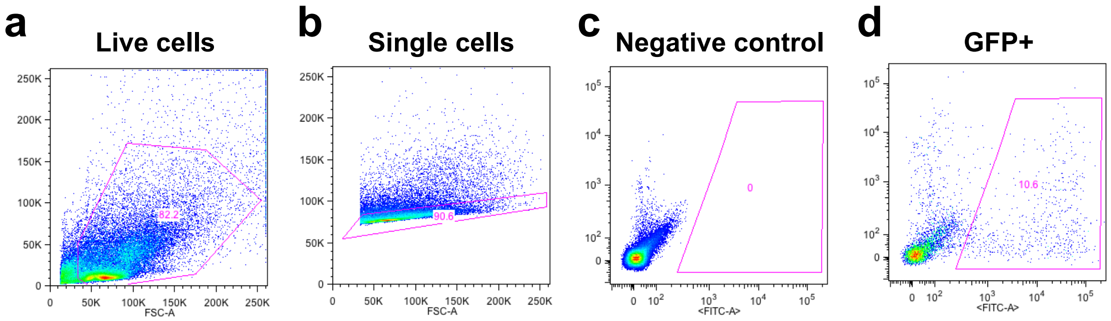

a
Live cells
b
Single cells
c
Negative control
d
GFP+
FSC-A
FSC-A
<FITC-A>
<FITC-A>
62.2
90.6
0
10.6

Supplementary Fig. 13 Gating strategy for the isolation of AT-GFP+ APCs by FACS

SV cells were first isolated from WAT depots of AT-GFP mice (n=3). (**a**) Live cells were selected by size on the basis of forward scatter (FSC) and side scatter (SSC). (**b**) Single cells were gated on both SSC and FSC width singlet's. (**c**) AT-GFP negative mice served as negative controls for gating. (**d**) Representative gating of GFP+ SVF cells isolated from AT-GFP control mice.

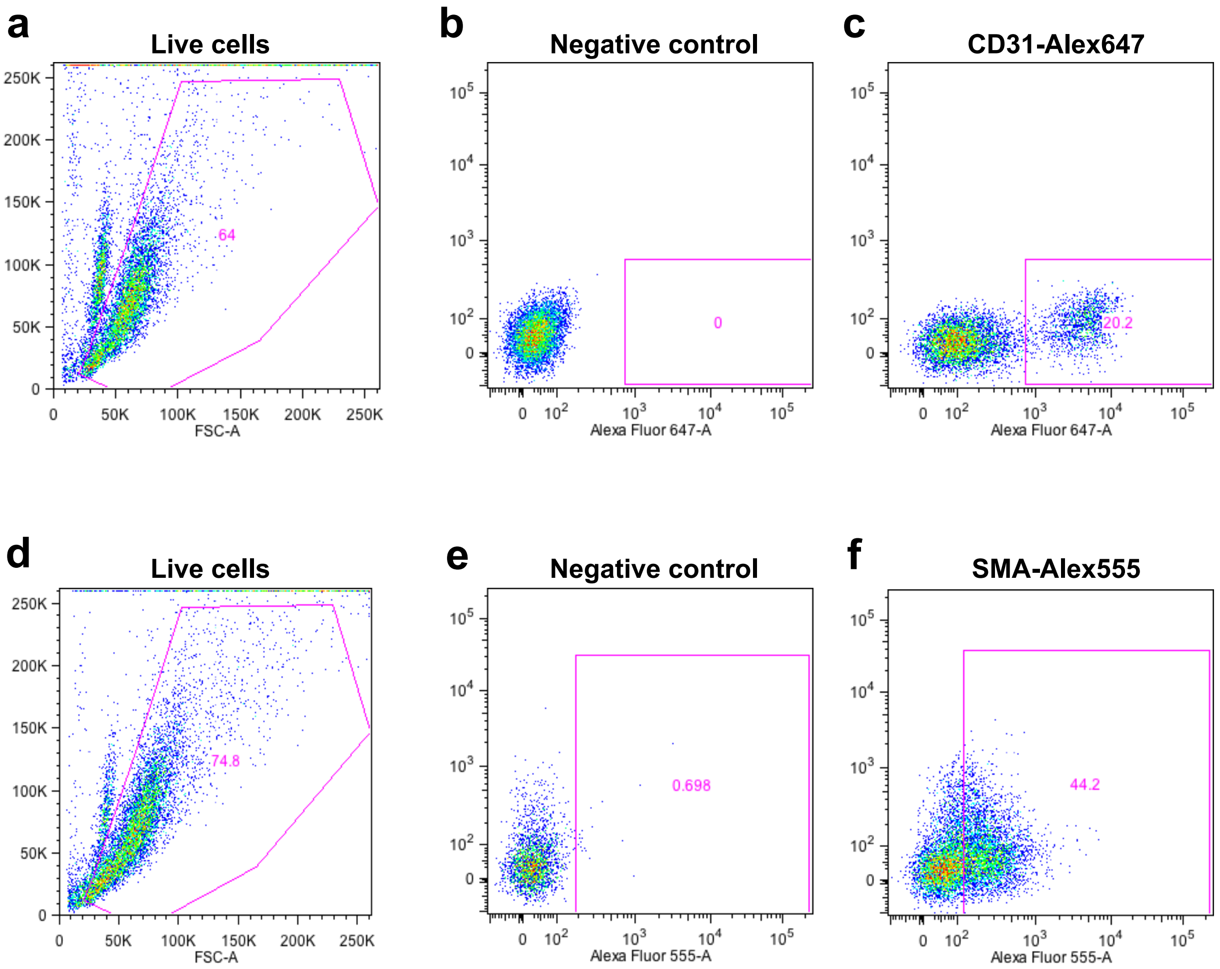

a
Live cells
64
FSC-A
b
Negative control
0
Alexa Fluor 647-A
c
CD31-Alex647
20.2
Alexa Fluor 647-A
d
Live cells
74.8
FSC-A
e
Negative control
0.698
Alexa Fluor 555-A
f
SMA-Alex555
44.2
Alexa Fluor 555-A

Supplementary Fig. 14 Gating strategy for flow analysis of CD31+ and SMA+ SVF cells

SV cells were first isolated from WAT depots of denoted mice. (**a**) live cells were selected by size on the basis of forward scatter (FSC) and side scatter (SSC). (**b**) Representative gating of donkey-anti-rat IgG Alex647 (match to Cy5) antibodies stained cells as negative control. (**c**) Representative gating of CD31 stained positive cells. (**d**) live cells were selected by size on the basis of forward scatter (FSC) and side scatter (SSC). (**e**) Representative gating of donkey-anti-rabbit IgG Alexa555 (match to Cy3) antibodies stained cells as negative control. (**f**) Representative gating of SMA stained positive cells.

Supplementary Table 1. Primers for Real-Time PCR analysis

Genes	Forward	Reverse
ACTA2	GTCCCAGACATCAGGGAGTAA	TCGGATACTTCAGCGTCAGGA
Adiponectin	TGTTCCTCTTAATCCTGCCCA	CCAACCTGCACAAGTTCCCTT
ADRP	GACCTTGTGTCCTCCGCTTAT	CAACCGCAATTTGTGGCTC
Ang1	CCAGGCCCGTTGTTCTTGAT	GGAAGGGAGACTTGCTCATTC
Ang2	AGAATAAGCAAGTCTCGCTTCC	TGAACCCTTTAGAGGCTCGGT
Angptl4	GTTTGCAGACTCAGCTCAAGC	CCAAGAGGTCTATCTGGCTCTG
aP2	AAGGTGAAGAGCATCATAACCCT	TCACGCCTTTCATAACACATTCC
Axud1	GTCTGTCCTCGGCTGTTGGAACC	CCACCTCAGCATCTCCAGCTTC
CD18	CAGGAATGCACCAAGTACAAAGT	CCTGGTCCAGTGAAGTTCAGC
CD31	ACGCTGGTGCTCTATGCAAG	TCAGTTGCTGCCCATTCATCA
CD34	AAGGCTGGGTGAAGACCCTTA	TGAATGGCCGTTTCTGGAAGT
CD54	CACAGTTCTCAAAGCACAGCG	GTGATGCTCAGGTATCCATCC
CD106	AGTTGGGGATTCGGTTGTTCT	CCCCTCATTCCTTACCACCC
CD144	CACTGCTTTGGGAGCCTTC	GGGGCAGCGATTCATTTTTCT
EGF	AGCATCTCTCGGATTGACCCA	CCTGTCCCGTTAAGGAAAACTCT
FGF2	GCGACCCACACGTCAAACTA	TCCCTTGATAGACACAACTCCTC
KLF2	CTCAGCGAGCCTATCTTGCC	CACGTTGTTTAGGTCCTCATCC
Leptin	GAGACCCCTGTGTCGGTTC	CTGCGTGTGTGAAATGTCATTG
Myh11	AAGCTGCGGCTAGAGGTCA	CCCTCCCTTTGATGGCTGAG
NG2 (CSPG4)	GGGCTGTGCTGTCTGTTGA	TGATTCCCTTCAGGTAAGGCA
PDGF-B	CATCCGCTCCTTTGATGATCTT	GTGCTCGGGTCATGTTCAAGT
PDGFRβ	AGGGGGCGTGATGACTAGG	TTCCAGGAGTGATACCAGCTT
Perilipin	GGGACCTGTGAGTGCTTCC	GTATTGAAGAGCCGGGATCTTTT
PPARγ	TCGCTGATGCACTGCCTATG	GAGAGGTCCACAGAGCTGATT
Rn18s	GTAACCCGTTGAACCCCATT	CCATCCAATCGGTAGTAGCG
VEGF-A	CTGCCGTCCGATTGAGACC	CCCCTCCTTGTACCACTGTC
VEGFR2	GCAGAAGATACTGTCACCACC	TTTGGCAAATACAACCCTTCAGA
